# Optimizing early neurological deterioration prediction in acute ischemic stroke patients following intravenous thrombolysis: a LASSO regression model approach

**DOI:** 10.3389/fnins.2024.1390117

**Published:** 2024-04-03

**Authors:** Ning Li, Ying-Lei Li, Jia-Min Shao, Chu-Han Wang, Si-Bo Li, Ye Jiang

**Affiliations:** ^1^Department of Neurology, Affiliated Hospital of Hebei University, Baoding, China; ^2^Department of Emergency Medicine, Baoding No.1 Central Hospital, Baoding, China

**Keywords:** acute ischemic stroke (AIS), intravenous thrombolysis (IVT), early neurological deterioration (END), LASSO regression, predictive modeling

## Abstract

**Background:**

Acute ischemic stroke (AIS) remains a leading cause of disability and mortality globally among adults. Despite Intravenous Thrombolysis (IVT) with recombinant tissue plasminogen activator (rt-PA) emerging as the standard treatment for AIS, approximately 6–40% of patients undergoing IVT experience Early Neurological Deterioration (END), significantly impacting treatment efficacy and patient prognosis.

**Objective:**

This study aimed to develop and validate a predictive model for END in AIS patients post rt-PA administration using the Least Absolute Shrinkage and Selection Operator (LASSO) regression approach.

**Methods:**

In this retrospective cohort study, data from 531 AIS patients treated with intravenous alteplase across two hospitals were analyzed. LASSO regression was employed to identify significant predictors of END, leading to the construction of a multivariate predictive model.

**Results:**

Six key predictors significantly associated with END were identified through LASSO regression analysis: previous stroke history, Body Mass Index (BMI), age, Onset to Treatment Time (OTT), lymphocyte count, and glucose levels. A predictive nomogram incorporating these factors was developed, effectively estimating the probability of END post-IVT. The model demonstrated robust predictive performance, with an Area Under the Curve (AUC) of 0.867 in the training set and 0.880 in the validation set.

**Conclusion:**

The LASSO regression-based predictive model accurately identifies critical risk factors leading to END in AIS patients following IVT. This model facilitates timely identification of high-risk patients by clinicians, enabling more personalized treatment strategies and optimizing patient management and outcomes.

## Introduction

Acute ischemic stroke (AIS) poses a significant global health challenge, being a leading cause of disability and mortality in adults worldwide. It inflicts a substantial burden not only on the affected individuals but also on societal and healthcare systems (Mendelson and Prabhakaran, 2021; Eren and Yilmaz, 2022; Liu et al., 2022). Intravenous thrombolysis (IVT), primarily with recombinant tissue plasminogen activator (rt-PA), has emerged as the standard therapeutic approach for AIS, markedly improving patient outcomes. However, approximately 6–40% of patients undergoing IVT experience early neurological deterioration (END) (Seners et al., 2014; Mao et al., 2022), which compromises treatment efficacy and can result in heightened morbidity and mortality.

The risk factors for Early Neurological Deterioration post-Intravenous Thrombolysis (END post-IVT) are diverse, encompassing hypertension, diabetes mellitus, previous stroke history, and various biochemical markers (Jin et al., 2023; Tian et al., 2023; Wang and Liu, 2023). Nevertheless, current predictive models for END following IVT have limitations, such as small sample sizes, regional constraints, or insufficient variable inclusion (Nair et al., 2022; Wang L. et al., 2022; Luo et al., 2023; Yang et al., 2023). Hence, there is a pressing need for more accurate models to aid clinicians in early identification of patients at high risk, facilitating timely therapeutic adjustments.

This study employs Least Absolute Shrinkage and Selection Operator (LASSO) regression, a robust method for variable selection, effectively reducing the number of predictors while maintaining model accuracy, thus enhancing interpretability and predictive power. Analyzing data from 531 AIS patients, our study not only investigates a multitude of potential risk factors but also constructs a predictive model based on LASSO regression, aimed at improving the precision of predicting END post-IVT.

Our research holds significant clinical relevance. Firstly, it is grounded in a relatively large patient sample, enhancing the representativeness and reliability of our findings. Secondly, the employed statistical methodology offers more precise guidance for clinical decision-making. Lastly, by identifying the most influential risk factors, our model can assist practitioners in effectively assessing patient risk in clinical practice and intervening promptly.

In summary, through advanced statistical techniques and comprehensive data analysis, this study aims to enhance the prediction of early neurological deterioration following intravenous thrombolysis in acute ischemic stroke, thereby facilitating more personalized and optimized treatment strategies for patients.

## Materials and methods

### Study design and participants

This study adhered to the recommendations of the Strengthening the Reporting of Observational Studies in Epidemiology (STROBE) guidelines for reporting observational research. This retrospective cohort study was conducted to develop and validate a predictive model for early deterioration in acute ischemic stroke patients post-alteplase intravenous thrombolytic treatment. Data were retrospectively gathered from Affiliated Hospital of Hebei University and Baoding First Central Hospital, spanning from July 2021 to October 2023. The objective was to identify pivotal predictors for early post-treatment deterioration. Inclusion Criteria: (1) Individuals aged 18 years or older. (2) Patients clinically diagnosed with acute ischemic stroke. (3) Administration of intravenous alteplase within 3 to 4.5 h from the onset of symptoms. Exclusion Criteria: (1) Patients who underwent endovascular treatment after receiving intravenous thrombolysis. (2) Lack of definitive focal hyperintensities in clinically relevant areas on initial or follow-up diffusion-weighted imaging. (3) Cases with incomplete patient data. (4) Patients with malignancies, autoimmune diseases, major organ failure, or active infections. This study was approved by the Institutional Review Boards of both the Affiliated Hospital of Hebei University and Baoding First Central Hospital, adhering to ethical standards for retrospective analysis. Due to the study’s retrospective nature, patient consent was waived, as approved by the IRBs. All patient data were anonymized to ensure confidentiality and compliance with ethical guidelines.

### Data collection

Data were collected retrospectively from patients with acute ischemic stroke who underwent intravenous alteplase thrombolysis, and they were subsequently randomly assigned to two groups: a training set and a validation set. This randomization ensured unbiased distribution of key clinical and demographic characteristics across both sets. Key variables included National Institutes of Health Stroke Scale (NIHSS) scores, gender, smoking and drinking habits, stroke history, diabetes mellitus, atrial fibrillation, coronary heart disease, hyperlipidemia, hypertension, systolic and diastolic blood pressure, body mass index, and age. Additionally, detailed lab parameters were recorded, such as white blood cell count, hemoglobin, platelet count, neutrophil count, and ratios involving platelets, neutrophils, lymphocytes, eosinophils, and monocytes. Metabolic factors like glucose, creatinine, uric acid, total cholesterol, triglycerides, low and high-density lipoprotein levels, and homocysteine were also included.

### Definition of END

In this study, END is defined as an increase of 2 or more points in the NIHSS score, assessed between the initial evaluation and a subsequent assessment conducted 24–36 h later (Siegler and Martin-Schild, 2011; Martin et al., 2019; Liu et al., 2023). This definition aims to precisely quantify and identify changes in neurological status, particularly in the critical early phase following thrombolytic treatment. By focusing on capturing subtle yet clinically significant shifts in NIHSS scores, this approach facilitates timely clinical intervention and management of patients post-thrombolysis.

### Statistical methodology

In developing the model for predicting END post-IVT, we transformed continuous variables into categorical ones, a strategy commonly embraced in risk prediction studies for enhanced interpretability and broader applicability (Bennette and Vickers, 2012; Barrio et al., 2017). This conversion, often essential for clinical datasets, simplifies interpretation and practical application in clinical environments. The determination of thresholds for these variables was facilitated using R software. When explicit cutoffs were not pre-established, variables were pragmatically divided into either binary or ternary categories. The frequencies and percentages of categorical variables were then tabulated. The χ^2^ tests or Fisher’s exact tests were employed for the analysis of categorical variables, depending on the data suitability. The selection of variables in the training dataset was carried out through LASSO regression, a method known for its efficiency in minimizing overfitting by choosing relevant predictors. The selection of the optimal lambda value followed the lambda.1se criterion, correlating with the most regularized model while staying within one standard error of the minimal cross-validation error. Subsequently, significant predictors identified through LASSO regression were included in a logistic regression model to determine independent predictors of END post-IVT. A nomogram incorporating these predictors was constructed to visually depict the risk factors and their respective weights in the prediction of END post-IVT. The performance of the nomogram was evaluated using Receiver Operating Characteristic (ROC) Curve analysis, specifically through the computation of the Area Under the Curve (AUC). Calibration plots, comparing predicted probabilities with actual outcomes, were generated to assess the model’s precision. Additionally, Decision Curve Analysis (DCA) was conducted to ascertain the clinical usefulness of the model by evaluating net benefits across various threshold probabilities. All statistical procedures were executed using R software (version 4.3.0), and a *p*-value less than 0.05 was regarded as indicative of statistical significance.

## Results

### Baseline characteristics

**FIGURE 1:**
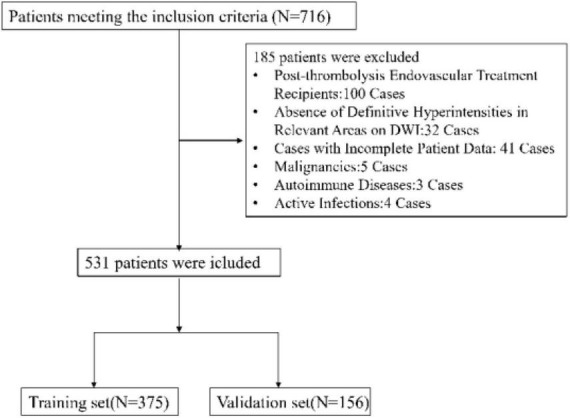
Flow chart of the study design. This figure illustrates the process of participant selection, data collection, and analysis phases for the retrospective cohort study. It details the inclusion and exclusion criteria and the division of participants into training and validation sets.

Between July 2021 and October 2023, our study initially involved 716 participants eligible for inclusion. Post-application of exclusion criteria, the cohort was narrowed to 531 patients for analysis, as illustrated in Figure 1. The group comprised 375 individuals in the training set and 156 in the validation set. Table 1 outlines the demographic and clinical attributes at baseline for both cohorts. This study incorporated the following 45 potential indicators related to END following intravenous thrombolysis: NIHSS, Gender (female or male), Smoking (Smoking habit presence), Drinking (Alcohol consumption habit), Stroke history, Diabetes mellitus, Atrial Fibrillation, Coronary heart disease, Hyperlipidemia, Hypertension, Systolic Blood Pressure, Diastolic Blood Pressure, Body Mass Index (BMI), Age, Onset to Treatment Time (OTT), Door to Needle Time (DNT), White Blood Cell Count, Hemoglobin, Platelet Count, Neutrophil Count, Platelet to Neutrophil Ratio, Neutrophil Percentage, Lymphocyte Count, Monocyte Count, Neutrophil to Lymphocyte Ratio, Monocyte to Neutrophil Ratio, Eosinophil Count, Neutrophil-to-Eosinophil Ratio, Eosinophil to Monocyte Ratio, High-Density Lipoprotein, Monocyte to HDL Ratio, Neutrophil to Lymphocyte Ratio, Systemic Immune Inflammation Index (SII), Prothrombin Time, Prothrombin Activity, Activated Partial Thromboplastin Time, International Normalized Ratio, Glucose, Creatinine, Uric Acid, Total Cholesterol, Triglycerides, Low-Density Lipoprotein, HDL to LDL ratio, Homocysteine. In this study, laboratory tests were categorized based on the specimen type required for analysis. Routine hematological assessments were conducted on whole blood, including White Blood Cell Count, Hemoglobin, Platelet Count, Neutrophil Count, etc. Biochemical parameters such as High-Density Lipoprotein (HDL), Glucose, Creatinine, Uric Acid, Total Cholesterol, Triglycerides, Low-Density Lipoprotein (LDL), and Homocysteine were analyzed using serum samples. Coagulation profiles, including Prothrombin Time, Prothrombin Activity, Activated Partial Thromboplastin Time, and International Normalized Ratio, were determined from plasma samples. Additionally, certain indicators such as the Neutrophil to Lymphocyte Ratio, Platelet to Neutrophil Ratio, and others, were derived from calculated values based on direct measurements. The uniformity observed in the 45 variables between the training and validation groups highlights the uniform nature of our study population. This similarity is vital as it implies that the model, developed from the training set data, has a high likelihood of being applicable to different patient groups. This aspect underscores the model’s strength in reliably predicting END post-IVT.

**TABLE 1:** Comparative analysis of potential predictive factors in a early neurological deterioration prediction in acute ischemic stroke patients following intravenous thrombolysis model between training and validation sets.

Variables	Total (*n* = 531)	Training set (*n* = 375)	Validation set (*n* = 156)	*p*
NIHSS, *n* (%)				0.445
0–5	333 (63)	229 (61)	104 (67)	
6–10	73 (14)	55 (15)	18 (12)	
>10	125 (24)	91 (24)	34 (22)	
Gender, *n* (%)				0.191
Female	176 (33)	125 (33)	62 (40)	
Male	355 (67)	250 (67)	94 (60)	
Smoking, *n* (%)				1
No	286 (54)	202 (54)	84 (54)	
Yes	245 (46)	173 (46)	72 (46)	
Drinking, *n* (%)				0.537
No	359 (68)	250 (67)	109 (70)	
Yes	172 (32)	125 (33)	47 (30)	
Stroke history, *n* (%)				0.538
No	457 (86)	320 (85)	137 (88)	
Yes	74 (14)	55 (15)	19 (12)	
Diabetes mellitus, *n* (%)				0.349
No	404 (76)	290 (77)	114 (73)	
Yes	127 (24)	85 (23)	42 (27)	
Atrial fibrillation, *n* (%)				0.45
No	466 (88)	326 (87)	140 (90)	
Yes	65 (12)	49 (13)	16 (10)	
Coronary heart disease, *n* (%)				1
No	409 (77)	289 (77)	120 (77)	
Yes	122 (23)	86 (23)	36 (23)	
Hyperlipidemia, *n* (%)				1
No	526 (99)	371 (99)	155 (99)	
Yes	5 (1)	4 (1)	1 (1)	
Hypertension, *n* (%)				1
No	201 (38)	142 (38)	59 (38)	
Yes	330 (62)	233 (62)	97 (62)	
Systolic blood pressure (mmHg), *n* (%)				0.916
<140	193 (36)	138 (37)	55 (35)	
140–159	231 (44)	161 (43)	70 (45)	
>159	107 (20)	76 (20)	31 (20)	
Diastolic blood pressure (mmHg), *n* (%)				0.829
<100	410 (77)	291 (78)	119 (76)	
≥100	121 (23)	84 (22)	37 (24)	
Body mass index, *n* (%)				0.417
<23.19	172 (32)	116 (31)	56 (36)	
23.19–26.37	169 (32)	125 (33)	44 (28)	
>26.37	190 (36)	134 (36)	56 (36)	
Age (years), *n* (%)				0.958
<60	195 (37)	139 (37)	56 (36)	
60–70	176 (33)	123 (33)	53 (34)	
>70	160 (30)	113 (30)	47 (30)	
OTT (min), *n* (%)				0.572
<150	178 (34)	124 (33)	54 (35)	
150–240	174 (33)	128 (34)	46 (29)	
>240	179 (34)	123 (33)	56 (36)	
DNT (min), *n* (%)				0.785
<40	181 (34)	125 (33)	56 (36)	
40–66	181 (34)	131 (35)	50 (32)	
>66	169 (32)	119 (32)	50 (32)	
White blood cell count (10 × 10^9^), *n* (%)				0.104
<6.50	185 (35)	141 (38)	44 (28)	
6.50–8.39	185 (35)	123 (33)	62 (40)	
>8.39	161 (30)	111 (30)	50 (32)	
Hemoglobin (g/L), *n* (%)				0.263
<110	180 (34)	119 (32)	61 (39)	
110–150	177 (33)	129 (34)	48 (31)	
>150	174 (33)	127 (34)	47 (30)	
Platelet count (×10^9^), *n* (%)				0.096
<192	185 (35)	139 (37)	46 (29)	
192–243	183 (34)	119 (32)	64 (41)	
>243	163 (31)	117 (31)	46 (29)	
Neutrophil count (×10^9^), *n* (%)				0.757
<3.77	183 (34)	132 (35)	51 (33)	
3.77–5.36	186 (35)	132 (35)	54 (35)	
>5.36	162 (31)	111 (30)	51 (33)	
Platelet to neutrophil ratio, *n* (%)				0.577
<39.44	170 (32)	117 (31)	53 (34)	
39.44–57.31	181 (34)	133 (35)	48 (31)	
>57.31	180 (34)	125 (33)	55 (35)	
Neutrophil percentage (%), *n* (%)				0.164
<57.2	185 (35)	127 (34)	58 (37)	
57.2–70.2	171 (32)	130 (35)	41 (26)	
>70.2	175 (33)	118 (31)	57 (37)	
Lymphocyte count (×10^9^), *n* (%)				0.491
<1.54	179 (34)	126 (34)	53 (34)	
1.54–2.27	178 (34)	131 (35)	47 (30)	
>2.27	174 (33)	118 (31)	56 (36)	
Monocyte count (×10^9^), *n* (%)				0.904
<0.45	192 (36)	136 (36)	56 (36)	
0.45–0.59	170 (32)	118 (31)	52 (33)	
>0.59	169 (32)	121 (32)	48 (31)	
Neutrophil to lymphocyte ratio, *n* (%)				0.306
<1.78	183 (34)	124 (33)	59 (38)	
1.78–3.27	175 (33)	131 (35)	44 (28)	
>3.27	173 (33)	120 (32)	53 (34)	
Monocyte to neutrophil ratio, *n* (%)				0.625
<0.10	184 (35)	126 (34)	58 (37)	
0.10–0.14	158 (30)	111 (30)	47 (30)	
>0.14	189 (36)	138 (37)	51 (33)	
Eosinophil count (×10^9^), *n* (%)				0.191
<0.07	188 (35)	140 (37)	48 (31)	
0.07–0.14	163 (31)	107 (29)	56 (36)	
>0.14	180 (34)	128 (34)	52 (33)	
Neutrophil-to-eosinophil ratio, *n* (%)				0.39
<30.5	190 (36)	135 (36)	55 (35)	
30.5–75	163 (31)	109 (29)	54 (35)	
>75	178 (34)	131 (35)	47 (30)	
Eosinophil to monocyte ratio, *n* (%)				0.586
<0.14	184 (35)	134 (36)	50 (32)	
0.14–0.27	166 (31)	118 (31)	48 (31)	
>0.27	181 (34)	123 (33)	58 (37)	
High-density lipoprotein (mmol/L), *n* (%)				0.946
<0.89	186 (35)	130 (35)	56 (36)	
0.89–1.14	180 (34)	127 (34)	53 (34)	
>1.14	165 (31)	118 (31)	47 (30)	
Monocyte to HDL ratio, *n* (%)				0.679
<0.42	171 (32)	119 (32)	52 (33)	
0.42–0.66	192 (36)	140 (37)	52 (33)	
>0.66	168 (32)	116 (31)	52 (33)	
Neutrophil to lymphocyte ratio, *n* (%)				0.564
<3.65	168 (32)	116 (31)	52 (33)	
3.65–5.88	178 (34)	123 (33)	55 (35)	
>5.88	185 (35)	136 (36)	49 (31)	
Systemic Immune Inflammation Index, *n* (%)				0.338
<496	176 (33)	122 (33)	54 (35)	
496–830	174 (33)	130 (35)	44 (28)	
>830	181 (34)	123 (33)	58 (37)	
Prothrombin time (s), *n* (%)				0.784
<10	190 (36)	137 (37)	53 (34)	
10–12	168 (32)	119 (32)	49 (31)	
>12	173 (33)	119 (32)	54 (35)	
Prothrombin activity (%), *n* (%)				0.954
<96	184 (35)	131 (35)	53 (34)	
96–106	175 (33)	124 (33)	51 (33)	
>106	172 (32)	120 (32)	52 (33)	
Activated partial thromboplastin time (s), *n* (%)				0.5
<26	172 (32)	126 (34)	46 (29)	
26–28	180 (34)	128 (34)	52 (33)	
>28	179 (34)	121 (32)	58 (37)	
International normalized ratio, *n* (%)				0.451
<0.90	179 (34)	126 (34)	53 (34)	
0.90–0.96	179 (34)	132 (35)	47 (30)	
>0.96	173 (33)	117 (31)	56 (36)	
Glucose (mmol/L), *n* (%)				0.349
<6.2	185 (35)	135 (36)	50 (32)	
6.2–8.0	170 (32)	113 (30)	57 (37)	
>8.0	176 (33)	127 (34)	49 (31)	
Creatinine (μmol/L), *n* (%)				0.877
<62	182 (34)	126 (34)	56 (36)	
62–75	177 (33)	126 (34)	51 (33)	
>75	172 (32)	123 (33)	49 (31)	
Uric acid (μmol/L), *n* (%)				0.858
<287	177 (33)	126 (34)	51 (33)	
287–369	176 (33)	126 (34)	50 (32)	
>369	178 (34)	123 (33)	55 (35)	
Total cholesterol (mmol/L), *n* (%)				0.122
<4.11	182 (34)	121 (32)	61 (39)	
4.11–4.97	173 (33)	120 (32)	53 (34)	
>4.97	176 (33)	134 (36)	42 (27)	
Triglycerides (mmol/L), n (%)				0.84
<1.05	182 (34)	126 (34)	56 (36)	
1.05–1.58	177 (33)	125 (33)	52 (33)	
>1.58	172 (32)	124 (33)	48 (31)	
Low density lipoprotein (mmol/L), *n* (%)				0.123
<2.27	181 (34)	118 (31)	63 (40)	
2.27–3.03	172 (32)	124 (33)	48 (31)	
>3.03	178 (34)	133 (35)	45 (29)	
HDL to LDL ratio, *n* (%)				0.155
<0.33	185 (35)	135 (36)	50 (32)	
0.33–0.45	174 (33)	128 (34)	46 (29)	
>0.45	172 (32)	112 (30)	60 (38)	
Homocysteine (μmol/L), *n* (%)				0.779
≤15	269 (51)	188 (50)	81 (52)	
>15	262 (49)	187 (50)	75 (48)	

### Variable selection

**FIGURE 2:**
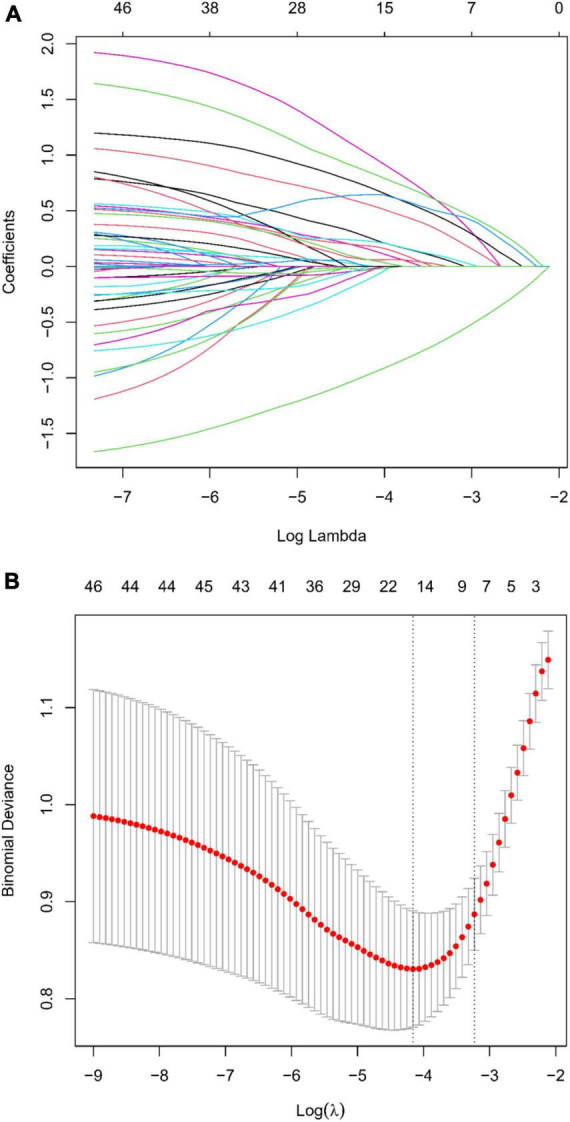
**(A)** LASSO regression coefficient profiles: displaying the progression of coefficients of various predictors as the regularization parameter (lambda) is increased. Each line represents a different predictor variable in the LASSO regression model. **(B)** Selection of Lambda in LASSO Regression: This graph shows the cross-validation curve for model tuning. The lambda value with the minimal cross-validation error is highlighted, indicating the optimal level of penalization for the LASSO model.

In our comprehensive investigation aimed at identifying variables critically influencing END post-IVT, we meticulously examined a dataset encompassing 45 variables. This dataset included a spectrum of demographic details, clinical histories, and extensive laboratory measurements. We employed the LASSO regression technique, renowned for its effectiveness in variable selection and its capability to prevent overfitting. This was executed using the glmnet package in R, incorporating a ten-fold cross-validation strategy to ascertain the optimal regularization parameter (λ). The choice of λ followed the one standard error rule based on the minimum criterion in cross-validation error. This approach was adopted to balance the model’s succinctness with its predictive precision (Figures 2A, B). Through this rigorous analytical procedure, we successfully narrowed down the array of factors to six key indicators that demonstrate a statistically significant association with END post-IVT. These pivotal indicators comprise Stroke History, BMI, Age, OTT, Lymphocyte Count, and Glucose, as well as the SII (Table 2). The variables retained, each manifesting non-zero coefficients in the LASSO model, were identified as crucial in unraveling the intricate interactions between systemic biological mechanisms and END post-IVT.

**TABLE 2:** Coefficients and lambda.1SE value of the LASSO regression.

Variable. Variable	Variable. Coefficient	Lambda.1SE
Stroke history	0.059599	0.03953
BMI	0.056924	
Age	0.033198	
OTT	0.09652	
Lymphocyte count	0.004739	
Glucose	0.085865	
SII	0.076369	

This table presents the outcomes of the LASSO regression analysis, which includes coefficients for key variables such as Stroke history, BMI, Age, OTT, Lymphocyte Count, Glucose, and SII. Additionally, it provides the lambda.1SE value, a crucial parameter in model selection that signifies the model’s robustness and predictive reliability. This analysis plays a pivotal role in the development of our predictive model, highlighting the significance of these variables in assessing the risk of early neurological deterioration (END) following intravenous thrombolysis (IVT) in acute ischemic stroke patients. BMI, body mass index; OTT, onset to treatment time; SII, Systemic Immune Inflammation Index.

### Multivariable analysis

In our multivariable logistic regression analysis, we discerned significant correlations between several key factors and the incidence of END post-IVT. These factors, initially identified through LASSO regression, included Stroke history, BMI, Age, OTT, Glucose, and the SII. These predictors, prominently correlated with increased risks, underscore vital demographic and biological elements that contribute to the occurrence of END in the aftermath of intravenous thrombolysis. These associations are comprehensively detailed in Table 3.

**TABLE 3:** Binary logistic regression analysis.

	*B*	SE	OR	CI	*Z*	*P*
Stroke history	1.25	0.538	3.5	1.5–8.0	2.65	0.008
BMI	0.85	0.337	2.3	1.1–4.8	2.02	0.013
Age	0.92	0.324	2.5	1.3–4.7	2.25	0.024
OTT	0.65	0.337	1.9	1.1–3.5	1.93	0.034
Glucose	1.1	0.323	3.0	1.6–5.6	2.7	0.007
SII	0.89	0.328	2.4	1.2–4.9	2.15	0.031

This table presents the outcomes of the binary logistic regression analysis conducted to identify potential predictors of early neurological deterioration following intravenous thrombolysis in acute ischemic stroke patients. This analysis initially included seven variables identified through LASSO regression. Of these, six were retained in the final prediction model, namely: Stroke history, BMI, Age, OTT, Glucose, SII. One variable, Lymphocyte Count, was excluded from the final model due to its *P*-value exceeding 0.05. BMI, body mass index; OTT, onset to treatment time; SII, Systemic Immune Inflammation Index.

### Development of a predictive nomogram

**FIGURE 3:**
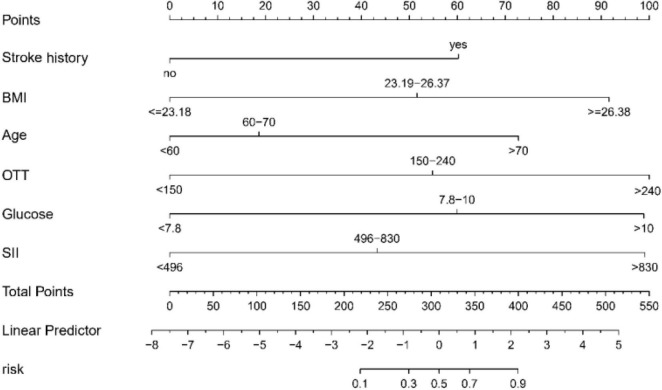
Predictive nomogram for early neurological deterioration (END) in acute ischemic stroke patients following intravenous thrombolysis (IVT): this nomogram integrates key predictive factors identified through LASSO regression, including stroke history, BMI, Age, OTT, Glucose, and SII, to estimate the probability of END post-IVT. BMI, body mass index; OTT, onset to treatment time; SII, Systemic Immune Inflammation Index.

Within the scope of this study, we crafted a sophisticated nomogram designed to estimate the probability of END post-IVT. This nomogram integrates six predictive factors that were meticulously identified: Stroke History, Body Mass Index (BMI), Age, Onset-to-Treatment Time (OTT), Glucose, and the Systemic Immune-Inflammation Index (SII), as illustrated in Figure 3. Each element contributes uniquely to a composite risk score, generated via the nomogram. This score directly corresponds to the potential likelihood of experiencing END. The application of this nomogram furnishes healthcare professionals with a precise, quantifiable instrument to evaluate risk. It further empowers them to customize patient care strategies with greater efficacy, thereby enhancing clinical decision-making processes.

### Validation of the predictive nomogram

**FIGURE 4:**
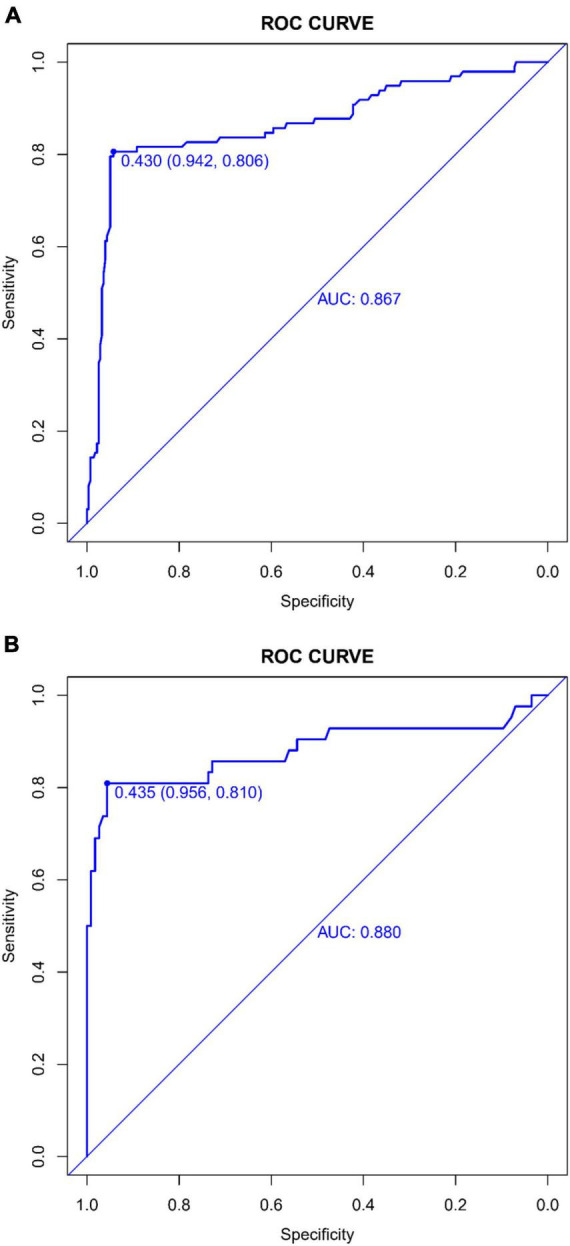
**(A)** Receiver operating characteristic (ROC) Curve of the predictive model in the training dataset: This figure depicts the ROC curve evaluating the performance of the predictive model for early neurological deterioration post-intravenous thrombolysis in the training dataset. It highlights the area under the curve (AUC) score, demonstrating the model’s discriminative capability. **(B)** Receiver operating characteristic (ROC) curve of the predictive model in the validation dataset: displayed here is the ROC curve assessing the efficacy of the predictive model for early neurological deterioration post-intravenous thrombolysis in the validation set. The area under the curve (AUC) value is provided as a measure of the model’s predictive accuracy.

**FIGURE 5:**
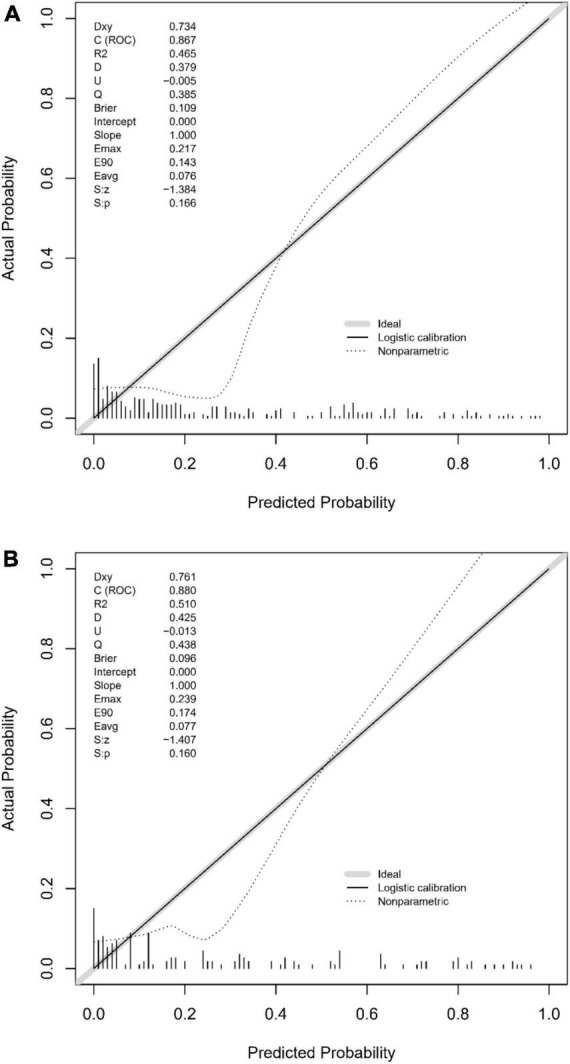
**(A)** Calibration plot for the training dataset: This figure demonstrates the calibration of the predictive model in the training set, comparing predicted probabilities of early neurological deterioration post-intravenous thrombolysis with observed outcomes. **(B)** Calibration plot for the validation dataset: comparison of predicted probabilities of early neurological deterioration post-intravenous thrombolysis with actual outcomes in the validation dataset, indicating the model’s calibration accuracy.

**FIGURE 6:**
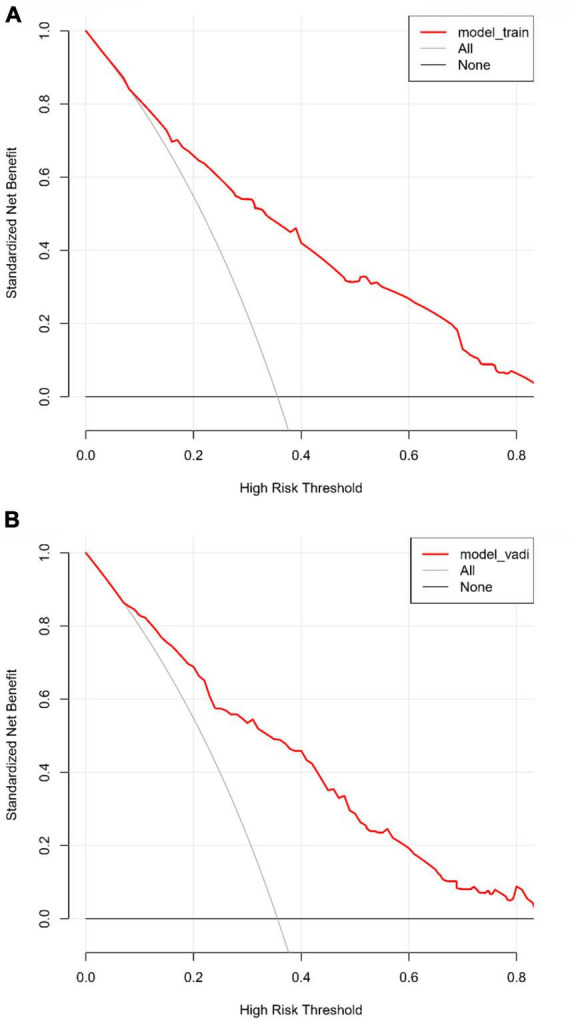
**(A)** Decision curve analysis (DCA) for the training dataset: this analysis illustrates the net benefits of the predictive model in the training set across different threshold probabilities. **(B)** Decision curve analysis (DCA) for the validation dataset: the plot shows the clinical usefulness of the model in the validation dataset by assessing net benefits at various probability thresholds.

The predictive accuracy of our developed nomogram for END post-IVT was rigorously evaluated. This assessment utilized the Area Under the Receiver Operating Characteristic (AUC-ROC) curve. In the training dataset, the nomogram exhibited a commendable AUC of 0.867 (95% Confidence Interval [CI]: 0.818–0.916), while the validation dataset demonstrated an even higher AUC of 0.880 (95% CI: 0.799–0.961). Such results underscore the nomogram’s exceptional discriminative prowess in both datasets, as depicted in Figures 4A, B. Additionally, the calibration curves for both the training (Figure 5A) and validation (Figure 5B) datasets showed good alignment with the ideal line, suggesting that the nomogram provides reasonably accurate estimations of END probabilities. The Decision Curve Analysis (DCA) conducted for both datasets further attests to the nomogram’s clinical value. The net benefit derived from the model substantially exceeds the outcomes of both the treat-all and treat-none strategies, as illustrated in Figures 6A, B. In practical application, the nomogram demonstrated high levels of sensitivity and specificity, reaffirming its robustness. Specifically, in the training set, we observed a sensitivity of 94.2% coupled with a specificity of 80.6% (Figure 4A). In the validation set, the sensitivity slightly increased to 95.6%, with the specificity maintaining a stable 81.0% (Figure 4B). Collectively, these metrics robustly validate the nomogram’s consistent and reliable performance, advocating for its potential integration into clinical practice for the effective risk stratification and management of patients at risk of END following intravenous thrombolysis.

## Discussion

This research offers critical insights into the prediction of END post-IVT in AIS patients. END, a common and severe complication post-thrombolysis, is closely linked to adverse long-term outcomes in AIS patients. Accurately predicting the occurrence of END is thus pivotal for clinical decision-making and patient management. By exhaustively analyzing a range of potential risk factors, our study develops a predictive model using the LASSO regression, enhancing our understanding of END risk factors and providing clinicians with an effective predictive tool to improve patient outcomes. A key innovation of our study lies in the application of LASSO regression, an efficient method for variable selection and reduction. LASSO regression is particularly valuable in handling extensive clinical data, as it eliminates unnecessary complexity and simplifies the model structure. This approach not only maintains the accuracy of the model but also improves its interpretability and clinical utility. By employing this technique, we were able to filter the most predictive variables out of a large pool of potential risk factors, offering a more precise and practical approach for the early identification and intervention of END post-IVT.

In our study, we developed a comprehensive predictive model integrating various factors closely associated with END post-IVT in patients with AIS. This model incorporates traditional stroke risk factors, such as age (Tschirret et al., 2018; Girot et al., 2020) and blood glucose levels (Simonsen et al., 2016; Huang et al., 2018; Meng et al., 2023), alongside emerging indicators like SII (Wei et al., 2023), providing a more holistic risk assessment. Specifically, the inclusion of past stroke history (Li et al., 2020; Jin et al., 2023) is pivotal as it may indicate an underlying state of cerebral vascular health in patients. Body mass index (BMI) (Tian et al., 2023) and onset-to-treatment time (OTT) (Meng et al., 2023; Shi et al., 2023) are also critical variables, reflecting the overall health condition of the patient and the timeliness of treatment, respectively. These factors are essential for predicting the efficacy of treatment. Notably, the integration of SII into our model offers a new perspective in understanding the complex biological processes associated with END. SII, as an indicator reflecting inflammatory and immune status, aids in delving deeper into the potential mechanisms underlying END. Overall, the amalgamation of these factors plays a vital role in enhancing the accuracy of predicting the risk of END in AIS patients following intravenous thrombolysis. This comprehensive approach is crucial for advancing our understanding and management of AIS, particularly in the context of post-thrombolysis outcomes.

Our study distinguishes itself by employing the LASSO regression, a technique not extensively explored in existing literature. The adaptability and efficacy of LASSO regression are well-documented across various medical research fields, demonstrating its utility in enhancing predictive modeling. Notably, its application ranges from predicting disease progression (Fujino et al., 2015) to diagnosing atypical conditions (Li et al., 2022) and assessing risk factors (McEligot et al., 2020; Wang Q. et al., 2022; Wong et al., 2023), underscoring its versatility and contribution to advancing personalized medicine. This method’s ability to refine the model by selecting the most relevant predictors offers an advantage over traditional logistic regression (Huang et al., 2018) and machine learning methods (Fan et al., 2023). Our model’s predictive accuracy, enhanced by LASSO regression, provides a more streamlined and clinically applicable tool for stroke management. Further, our research contributes to personalized medicine in stroke care. By integrating a diverse range of clinical and biochemical parameters, our predictive model addresses the heterogeneity inherent in AIS presentations, a theme echoed in recent studies (Jin et al., 2023; Luo et al., 2023). In conclusion, our research not only corroborates with existing findings regarding END post-IVT but also introduces an advanced predictive model. This model’s methodological sophistication and potential for clinical applicability underscore its significance in the landscape of stroke management. Our findings pave the way for more accurate, efficient, and individualized risk stratification in AIS patients undergoing intravenous thrombolysis.

The predictive model developed in this study offers substantial clinical utility in predicting and managing the risk of END post-IVT. Integrating vital clinical and laboratory parameters, this model provides a quantified risk score, enhancing physicians’ ability to determine the most appropriate treatment strategies. Its simplicity and intuitive design ensure easy implementation in clinical settings, utilizing readily available standard clinical data such as medical history, vital signs, and laboratory results. This model plays a crucial role in the clinical decision-making process by offering a quantitative tool for individualized patient risk assessment, thereby enabling physicians to identify high-risk patients and tailor treatment plans accordingly. Furthermore, it aids in optimizing resource allocation, directing more monitoring and intervention resources toward patients at greater risk of END post-IVT. Additionally, the model serves as an invaluable tool for patient education and communication, facilitating discussions between physicians, patients, and their families about disease risks and prognosis, and promoting patient participation in the decision-making process.

Building upon our current model’s foundations, the inclusion of cerebral artery occlusion location as a variable represents a promising direction for future enhancements. Recognizing the significant influence of occlusion site, particularly for M2 or more distal occlusions (Broccolini et al., 2023), on the risk of END opens avenues for refining our predictive capabilities. This consideration aligns with the growing demand for precision medicine, where understanding the intricate relationship between specific stroke characteristics and patient outcomes can lead to more tailored and effective interventions. In light of this, future iterations of our model will explore the feasibility and impact of incorporating occlusion location data, striving to offer an even more comprehensive tool for stroke risk assessment. This evolution reflects our commitment to continually advancing stroke management practices, ensuring that our predictive model remains at the forefront of aiding clinicians in delivering personalized care to AIS patients.

While this study provides important insights into the prediction of END post-IVT, it is not without limitations. Firstly, despite a relatively large sample size, our research was primarily based on data from two hospitals in a specific region, which may limit the generalizability of our findings. Additionally, the retrospective design of the study could introduce potential biases such as selection bias and information bias. Therefore, our findings necessitate further validation in prospective, multicenter studies.

For future research, we recommend expanding the sample size and diversifying the study population. This could be achieved by including patients from various geographic locations and different healthcare settings, thereby enhancing the universality and transferability of the model. Furthermore, future studies should consider incorporating additional potential predictive factors, such as genetic markers and lifestyle factors, to improve the predictive accuracy and clinical relevance of the model. Moreover, future research should explore the applicability of the model in different treatment scenarios, such as the combination of acute phase intravenous thrombolysis with interventional therapies. Assessing the practical effectiveness of the model in real-world clinical settings is also crucial, for instance, through clinical trials evaluating the model’s efficacy and its impact on patient outcomes.

In summary, our study advances the prediction of END post-IVT in acute ischemic stroke. The key finding is a robust LASSO regression-based predictive model that efficiently identifies patients at high risk for END using clinical and laboratory data. This model offers practical utility for personalized patient management and resource optimization in stroke care. While promising, further research is needed to validate its applicability across diverse clinical settings. Our work lays the groundwork for enhancing stroke treatment strategies and underscores the importance of continuous research in this domain.

## Data availability statement

The original contributions presented in this study are included in the article/supplementary material, further inquiries can be directed to the corresponding author.

## Ethics statement

The studies involving humans were approved by the Institutional Review Board of the Affiliated Hospital of Hebei University and the Institutional Review Board of the Baoding First Central Hospital. The studies were conducted in accordance with the local legislation and institutional requirements. The ethics committee/institutional review board waived the requirement of written informed consent for participation from the participants or the participants’ legal guardians/next of kin because of the retrospective nature of the study.

## Author contributions

NL: Conceptualization, Data curation, Formal analysis, Investigation, Methodology, Project administration, Resources, Software, Validation, Visualization, Writing – original draft, Writing – review and editing. Y-LL: Conceptualization, Data curation, Formal analysis, Investigation, Methodology, Resources, Software, Validation, Visualization, Writing – original draft, Writing – review and editing. J-MS: Conceptualization, Data curation, Formal analysis, Investigation, Methodology, Software, Writing – original draft. C-HW: Conceptualization, Data curation, Formal analysis, Investigation, Methodology, Software, Writing – original draft. S-BL: Conceptualization, Formal analysis, Investigation, Methodology, Writing – review and editing. YJ: Formal analysis, Investigation, Methodology, Project administration, Supervision, Validation, Visualization, Writing – original draft, Writing – review and editing.
